# Rationalizing Patterns in the Cation Ordering, Geometric Distortion, and Electronic Properties of a Class of I–V–VI_2_ Chalcogenide Semiconductors

**DOI:** 10.1021/acs.jpcc.5c05853

**Published:** 2026-01-12

**Authors:** Gabe Flanagin, Robert F. Berger

**Affiliations:** Department of Chemistry, 1632 Western Washington University, Bellingham Washington 98225, United States

## Abstract

Chalcogenide semiconductors are of interest as light absorbers for solar energy conversion applications. To fully realize their potential, it is crucial to understand how to tune the optoelectronic properties of these compounds through changes in their composition and atomic structure. In this paper, we use density functional theory calculations as a guide to understanding and tuning a class of ABX_2_ (I–V–VI_2_) compounds (A = Li, Na, K, Rb; B = As, Sb, Bi; X = S, Se). While these compounds can all be viewed as superstructures of NaCl, they have remarkable variety in their patterns of cation ordering and geometric distortion. By exploring a novel model that connects the sequences of atoms along B–X bond axes to local bonding motifs (2-center 2-electron and 3-center 4-electron), we rationalize the structural variety in this class of compounds and suggest how it can be leveraged to tune their properties such as band gap.

## Graphical abstract

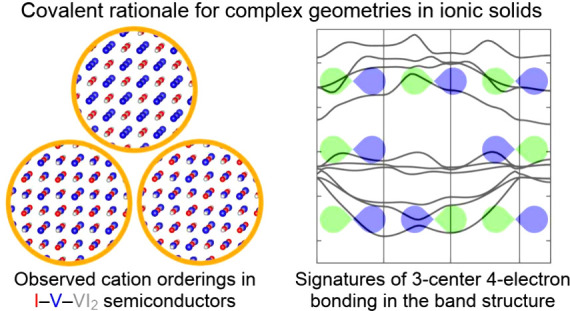

## Introduction

In the development of solar energy conversion technologies, it is crucial to identify classes of light-absorbing semiconductors whose composition and atomic structure can be tuned. Computationally rationalizing the structural and electronic features of these materials can unlock the potential to optimize their properties and performance. In recent years, lead-halide perovskites^1–3^ have been one particularly notable class of tunable materials. Since their emergence in 2009,^4^ perovskite photovoltaic efficiencies have steadily improved to match silicon-based technologies,^5^ driven in large part by compositional and structural tuning of the underlying materials. Similarly, various families of chalcogenide semiconductors have been developed and tuned for solar energy conversion.^6–9^ The optoelectronic tunability of these classes of compounds benefit from their flexible composition at all sites, and often other degrees of structural freedom that can be accessed via layering,^10–12^ strain,^13–17^ and/or defects.^18–20^

Our focus in this paper is on a class of chalcogenide semiconductors with the general formula ABX_2_, where A is an alkali metal cation, B is a pnictogen cation, and X is a chalcogenide anion. These compounds have electron configurations related to lead-halide perovskites, in that their B-site cations (As^3+^, Sb^3+^, or Bi^3+^) are isoelectronic with Pb^2+^ and their X-site anions (S^2–^ or Se^2–^) are isoelectronic with halide anions. To date, some research has focused on the crystal structure and applications of individual compounds within this ABX_2_ class. For example, in addition to papers that have reported the crystal structures of these compounds,^21–29^ work on the atomic and electronic structure of AAsS_2_ ^26^ and AAsSe_2_ ^27^ compounds has noted relationships between composition and band gap, and NaSbS_2_ has been explored as an emerging solar absorber.^30–33^

To our knowledge, however, previous work has not aimed to rationalize a remarkable feature of this class of ABX_2_ compounds as a whole: their structural diversity. While all can be viewed as cation-ordered variants of the NaCl structure, they adopt a variety of distortions and patterns of cation ordering when elements are substituted at each site. In this paper, we aim to provide a more complete view of the similarities and differences in atomic and electronic structure within this class of materials. In doing so, we demonstrate the importance of both ionic and covalent driving forces in understanding their structural variety, highlight key features in their electronic structure and bonding, and identify opportunities to use this knowledge to tune their properties. Notably, we show that the variety in cation ordering can be rationalized by focusing on the sequences of atoms along individual bond axes, and trends in band gap with A-site size are highly dependent on B-site identity and coordination.

## Computational Methods

All calculations in this work are performed within density functional theory (DFT) using the VASP package^34–37^ and PAW potentials^38^ (details in the Supporting Information). For ground-state geometries and structural energies, the PBE functional (a generalized gradient approximation) is used for its proven ability to capture energetic differences among competing structural phases.^39^ To consider the possibility that relative structural energetics are affected by van der Waals forces, we provide additional tests (in the Supporting Information) which include van der Waals corrections using the D3 method of Grimme.^40,41^ We find that van der Waals corrections do not qualitatively change any of the reported trends.

To calculate electronic band gaps in a manner more predictive of experimental values, these PBE-optimized compounds are then computed using the HSE06 hybrid functional.^42^ Images of band structures and (projected) densities of states are nonetheless computed using PBE, as our focus in those images is on qualitative trends in band energy and character, which result from orbital symmetry and are therefore sufficiently captured by PBE calculations. Results in the body of the paper do not include spin–orbit coupling.^43^ Based on our own testing of HSE06 calculations that include spin–orbit coupling (shown in the Supporting Information) and past computational work on similar compounds by others,^44^ the effect of spin–orbit coupling on the band gaps of these compounds is relatively small and does not qualitatively change trends.

All geometry optimizations and band gaps are computed using a Γ-centered *k*-point mesh, with the density of *k*-points chosen to match a cubic NaCl-type (8-atom) unit cell with 6 × 6 × 6 *k*-points. That is, as the NaCl-type superstructures described in this work typically require larger unit cells, proportionally fewer *k*-points are used to maintain the same level of precision. A plane-wave basis set cutoff of 400 eV is used throughout. Tests of NaAsS_2_, NaSbS_2_, and RbBiS_2_ provided in the Supporting Information show that the computed structural energies and band gaps do not change significantly when the number of *k*-points or basis set cutoff increase. Images of crystal structures are produced using the VESTA visualization program.^45^ Band structures and (projected) densities of states are plotted using the Sumo toolkit.^46^ Throughout this work, the Materials Project database^47^ is used as a valuable source of information regarding experimentally existing compounds and their crystal structures.

## Results and Discussion

### Existing Variety of Compounds

Of the broader class of compounds we consider (A = Li, Na, K, Rb; B = As, Sb, Bi; X = S, Se), the structures of existing, cation-ordered NaCl-type phases are summarized in Table 1. When viewed through the lens of ionic bonding, all of these phases are superstructures of NaCl, in which the X^2–^ anions occupy the chloride sites and the A^+^ and B^3+^ cations occupy the sodium sites. As in NaCl itself, the alternating arrangements of cations and anions lead to favorable Coulombic potential energy. Somewhat mysteriously, however, the precise ordering of A^+^ and B^3+^ cations varies significantly with changing elements. As the third column of the table shows, the cation arrangement in some cases forms layers in high-symmetry crystallographic directions, and in other cases is more complex.

**Table 1:** Summary of the Structures of Existing, Cation-Ordered NaCl-Type Phases with the Chemical formula ABX_2_ (A = Li, Na, K, Rb; B = As, Sb, Bi; X = S, Se)

Chemical formula and reference(s)	Space group	Orientation of A and B layers relative to NaCl structure	Nearest-neighbor coordination around B
LiAsS_2_ ^26^	*Cc*	001	Trigonal pyramidal
LiAsSe_2_ ^27^	*Cc*	001	Trigonal pyramidal
LiSbS_2_ ^28^	*C*2/*c*	Not a simple layering	Seesaw
NaAsS_2_ ^23,24^	*P*2_1_/*c*	001	Trigonal pyramidal
NaAsSe_2_ ^27^	*Pc*	Not a simple layering	Trigonal pyramidal
	*Pbca*	001	Trigonal pyramidal
NaSbS_2_ ^25^	*C*2/*c*	Not a simple layering	Seesaw
KSbS_2_ ^22^	*C*2/*c*	Not a simple layering	Seesaw
KBiS_2_ ^29^	*R*3̅*m*	111	Octahedral
KBiSe_2_ ^29^	*R*3̅*m*	111	Octahedral
RbBiS_2_ ^21^	*R*3̅*m*	111	Octahedral

While all cations in the NaCl structure are octahedrally coordinated by anions, most compounds in Table 1 are heavily distorted around the B site to break this octahedral symmetry. When viewed through the lens of covalent bonding, the distortions in these phases facilitate the types of B-site coordination that one would expect in main-group molecules. In each of the B = As compounds, rather than adopting true octahedral coordination among its neighboring sulfur atoms, the arsenic atoms instead shift their positions closer to three of their neighbors and farther from the other three, adopting trigonal pyramidal coordination as a nitrogen atom would. Farther down the pnictogen column of the periodic table, antimony adopts seesaw coordination and bismuth adopts octahedral coordination, as is often seen in molecules with increasingly large central atoms. Though we need not cover it in detail here, molecular chemists’ views of such “hypervalency” have a long and contentious history, and have evolved over the years. While seesaw and octahedral coordination were previously seen as exceptions to the octet rule involving *d* orbitals in the hybridization of the central atom^48^ (a view that has fallen out of favor), such geometries are now typically rationalized within the framework of the octet rule, based on 3-center 4-electron bonding.^49^

While the overall NaCl framework of these compounds and their B-site coordination can be understood based on their ionic and covalent character, respectively, the variety in their cation ordering is not as easily understood. For example, why do B-site arsenic atoms tend to reside in 001-oriented layers, while B-site bismuth atoms tend to reside in 111-oriented layers? We explore questions such as these in the next section.

### Computation of Structural Energetics

To begin to probe the driving forces for energetic stability in this class of compounds, we focus on three phases: the experimentally observed structures of NaAsS_2_ (*P*2_1_/*c*, Figure 1a), NaSbS_2_ (*C*2/*c*, Figure 1b), and RbBiS_2_ (*R*3̅*m*, Figure 1c). We choose this sampling of three phases because, though not a complete enumeration of known phases, they represent the existing variety in B-site coordination. B-site atoms have trigonal pyramidal coordination in NaAsS_2_, seesaw coordination in NaSbS_2_, and octahedral coordination in RbBiS_2_. For the clarity of our discussion, we do not include calculations of other closely related known phases (e.g., the *Cc* phase of LiAsS_2_ ^26^ and LiAsS_2_ ^27^), whose structure and electronic properties are extremely similar to one of the phases computed.

**Figure 1:**
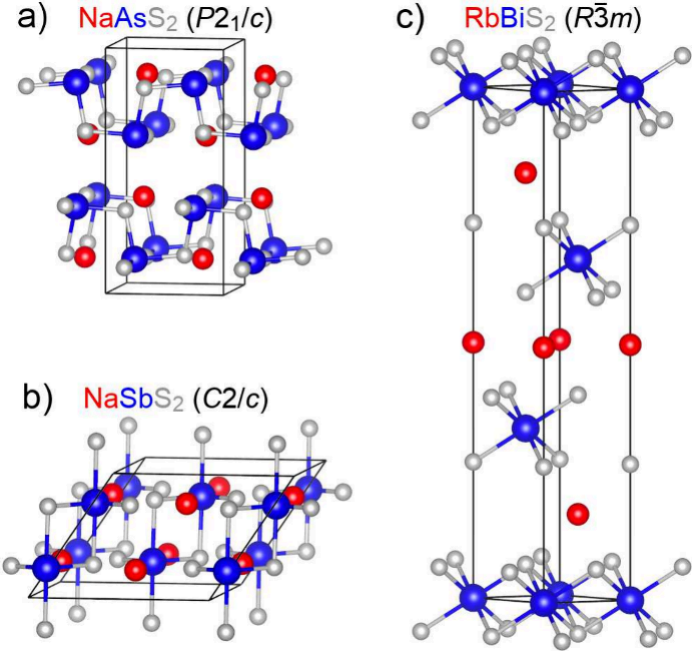
Three experimentally observed ABX_2_ phases that are studied computationally in this work, with nearest-neighbor B–X bonds shown. The crystal structures adopted by (a) NaAsS_2_ (*P*2_1_/*c*), (b) NaSbS_2_ (*C*2/*c*), and (c) RbBiS_2_ (*R*3̅*m*) can be viewed as superstructures of NaCl, each with a distinct pattern of distortion and cation ordering.

It should be noted that, by taking the approach of comparing the structural energies of these three phases, we are not formally tackling the question of whether cation-ordered NaCl-type ABX_2_ phases are global minima for these combinations of elements to begin with. Indeed, there are examples in which ABX_2_ phases have been synthesized with either cation-disordered structures (e.g., NaBiS_2_ and NaBiSe_2_)^29^ or with structures less closely related to NaCl (e.g., KAsSe_2_ and RbAsSe_2_).^50^ Still, because many of these cation-ordered NaCl-type ABX_2_ phases have been synthesized (Table 1) and still more could likely be accessed in the future, there is value in considering the three phases shown in Figure 1 for a broad set of elemental compositions.

Computations of the structural energies of these three competing phases are summarized in Figure 2. For each combination of elements in the figure, the structural energy per atom of the RbBiS_2_ structure is defined to be the zero-energy baseline, with the energies of the NaAsS_2_ and NaSbS_2_ structures reported relative to that. Therefore, a negative energy indicates that a NaAsS_2_- or NaSbS_2_-type phase is energetically preferred over the corresponding RbBiS_2_-type phase, while a positive energy indicates that a RbBiS_2_-type phase is energetically preferred. The results are generally consistent with chemical intuition and experimental knowledge of existing phases. As one would expect based on the known structures of existing compounds and the typical coordination of arsenic, antimony, and bismuth in molecules, the RbBiS_2_ structure is most stable for all B = Bi compositions, and the NaSbS_2_ structure is most stable for all B = Sb compositions. For B = As compositions, the NaAsS_2_ and NaSbS_2_ structures are in close energetic competition. While some of the B = As compounds (LiAsS_2_, NaAsS_2_, and KAsS_2_) show the expected preference for trigonal pyramidal coordination, others (RbAsS_2_, LiAsSe_2_, NaAsSe_2_, KAsSe_2_, and RbAsSe_2_) show a slight preference (0.008 eV per atom on average) for seesaw coordination. Given that the energy differences in these latter cases are small, and indeed disagree with the experimental preferences for trigonal pyramidal coordination in LiAsSe_2_ and NaAsSe_2_ cited in Table 1, it is possible that these discrepancies simply highlight a small dependence on the computational methods chosen.

**Figure 2:**
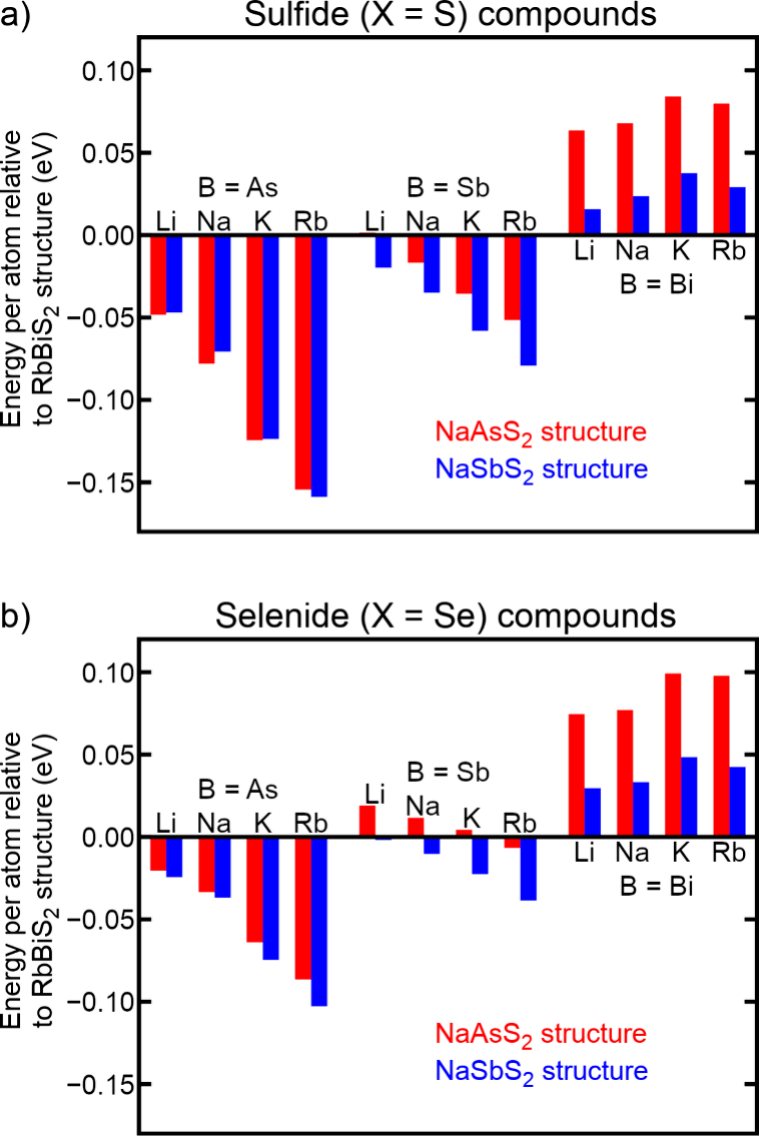
Comparisons of the DFT-PBE-computed structural energy per atom of (a) sulfide and (b) selenide compounds in their geometry-optimized NaAsS_2_, NaSbS_2_, and RbBiS_2_ crystal phases. For each combination of elements, the structural energy per atom of the RbBiS_2_ structure is defined to be the zero-energy baseline, with the energies of the NaAsS_2_ (red) and NaSbS_2_ (blue) structures reported relative to that.

These findings are not significantly affected by our subsequent calculations of phonon modes and frequencies of the most stable phase of each stoichiometry. Of the 24 combinations of elements considered, four (LiAsSe_2_, LiSbSe_2_, KAsSe_2_, and RbSbS_2_) have imaginary phonon frequencies, suggesting possible distortions. However, all of those frequencies are less imaginary than those in, for example, the cubic perovskite SrTiO_3_, whose distortions are known to be suppressed well below room temperature.

### Bond Axes as a Primary Driver of Cation Ordering

While it is not surprising that arsenic, antimony, and bismuth atoms would prefer different nearest-neighbor coordination environments, it is not immediately clear why this would lead to different patterns of cation ordering in these ABX_2_ compounds. To rationalize the variety of cation ordering patterns, we next focus on chemical bonding along the individual B–X bond axes. To highlight solely the differences in cation ordering, the structures of NaAsS_2_, NaSbS_2_, and RbBiS_2_ are shown on the left side of Figure 3 in their undistorted forms – that is, as true superstructures of NaCl. The right side of Figure 3 highlights the atoms along the three perpendicular bond axes surrounding a B atom. Note that all B atoms are symmetry-equivalent in each of these structures, so the choice of central atom is arbitrary. It is clear in these pictures that two types of bond axes emerge: some in which all cations are B (···–X–B–X–B–X–···, which we define as Type 1), and others in which cations alternate between A and B (···–X–A–X–B–X–···, which we define as Type 2). The three structure types differ in how these types of axes are distributed. In NaAsS_2_ (Figure 3a), all three B–X bond axes are Type 1. In NaSbS_2_ (Figure 3b), two are Type 1 and one is Type 2. In RbBiS_2_ (Figure 3c), all three axes are Type 2.

**Figure 3:**
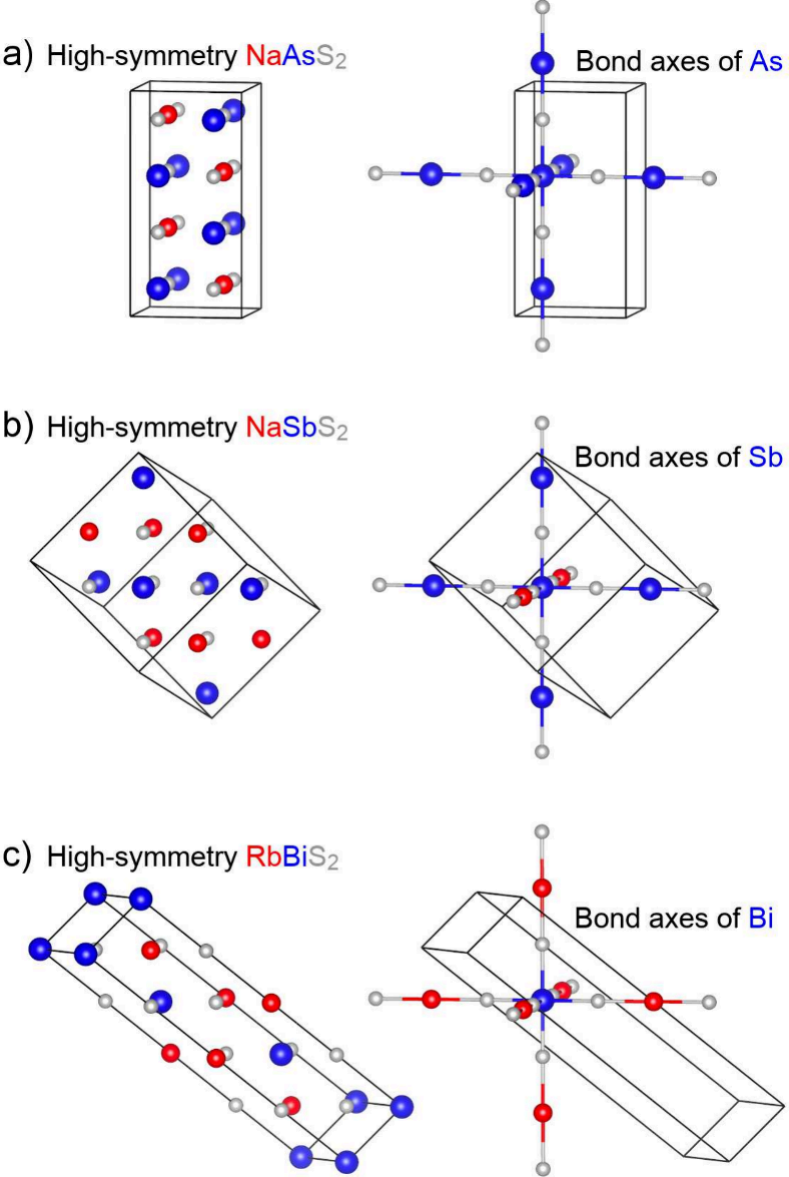
Experimentally observed cation orderings of (a) NaAsS_2_, (b) NaSbS_2_, and (c) RbBiS_2_, with atomic positions constrained to the high-symmetry positions of the NaCl structure. On the left, all atoms are shown separately. On the right, the three perpendicular bond axes surrounding a B-site atom are emphasized, highlighting the distinct cation ordering patterns in each structure.

Along each B–X axis, chemical bonding primarily involves interactions among the B and X valence *p* orbitals oriented along that axis. As shown in Figure 4, the two different types of bond axes lend themselves to different distortions and patterns of covalent bonding. For simplicity in electron counting, we assume ionic charges of B^3+^ (in which each valence *p* orbital carries zero electrons) and X^2–^ (in which each valence *p* orbital carries two electrons). Along Type 1 axes (Figure 4a), energy is minimized through the formation of B–X pairs—that is, each atom sits closer to one of its neighbors and farther from the other. In this situation, each B–X pair forms a typical 2-center 2-electron covalent bond. In contrast, along Type 2 axes (Figure 4b), there are two X^2–^ anions for each B^3+^ cation. These axes therefore feature X–B–X motifs, each of which forms a 3-center 4-electron bond as seen in many main-group molecules with large central atoms.

**Figure 4:**
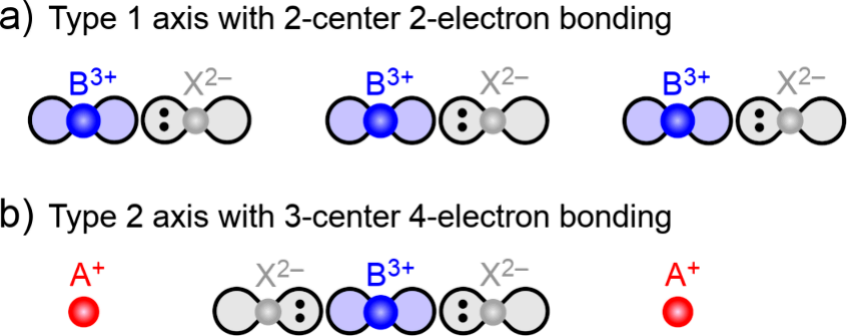
Schematic illustrations of B–X covalent bonding along the two distinct types of bond axes surrounding a B-site atom. a) Along a Type 1 axis (···–X–B–X–B–X–···), B–X pairs are positioned to form 2-center 2-electron bonds. b) Along a Type 2 axis (···–X–A–X–B–X–···), X–B–X motifs are positioned to form 3-center 4-electron bonds.

As Figure 4 shows, a B atom is bonded to one X atom along each Type 1 axis and two X atoms along each Type 2 axis. As a result, arsenic atoms in NaAsS_2_ (in which all axes are Type 1) have trigonal pyramidal coordination, antimony atoms in NaSbS_2_ (in which two axes are Type 1 and one is Type 2) have seesaw coordination, and bismuth atoms in RbBiS_2_ (in which all axes are Type 2) have octahedral coordination. We note that these coordination environments – trigonal pyramidal for arsenic, seesaw for antimony, and octahedral for bismuth – follow the typical trends from lower to higher coordination as the central atom is further down the periodic table. We now see that the sequence of atoms and consequent bonding along each B–X axis is a primary reason for the variety of cation orderings in I–V–VI_2_ compounds.

### Evidence for 3-Center 4-Electron Bonds in the Electronic Band Structure

Though intuitively plausible, our descriptions of covalent bonding along each type of bond axis in the previous subsection have not yet been supported by evidence. We now turn to computed band structures, projected densities of states, and electron localization functions in order to more quantitatively identify signatures of the bonding described above.

Figure 5 shows various views of the valence and conduction bands of RbBiS_2_. Figure 5a, in addition to showing the band energies and total density of states (black curve), highlights the contribution of Bi *p* orbitals to the bands (both in the blue tint within the band structure and the blue projected density of states curve). Similarly, Figure 5b highlights the contribution of S *p* orbitals in yellow. To some degree, these images support the traditional ionic view of this compound. Bi *p* orbitals contribute most significantly to the three lowest-energy conduction bands, consistent with the fact that the one Bi^3+^ cation in the primitive unit cell of RbBiS_2_ has three unfilled 6*p* orbitals. S *p* orbitals contribute most significantly to the six highest-energy valence bands, consistent with the fact that the two S^2–^ anions have a total of six filled 3*p* orbitals.

**Figure 5:**
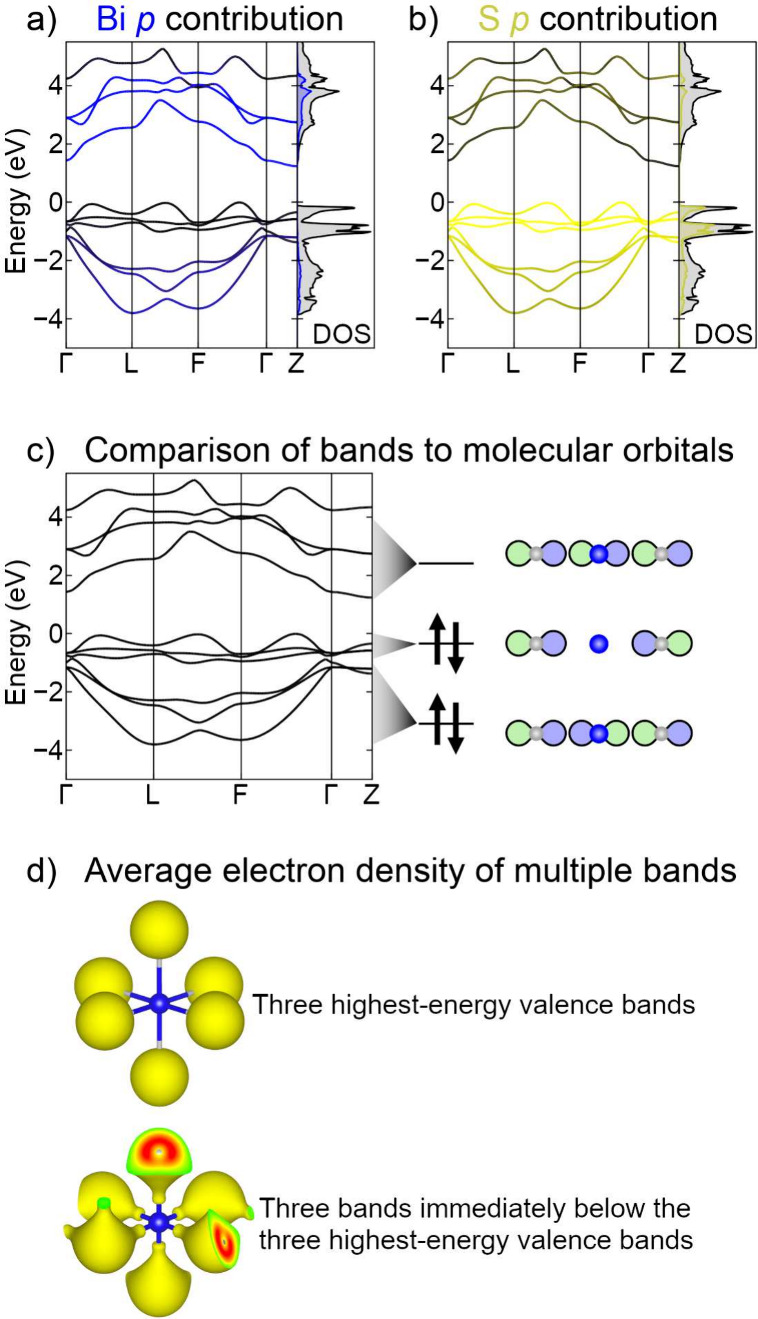
Various views of the DFT-PBE-computed valence and conduction bands of RbBiS_2_. (a) Contributions of Bi *p* states to the total are shown both in the blue tint within the band structure and the blue projected density of states curve. (b) Contributions of S *p* states to the total are similarly shown in yellow. (c) Based on the contributions of Bi *p* and S *p*, it can be inferred that groups of three bands carry the hallmarks of the three molecular orbitals in a 3-center 4-electron bond. (d) Images of the computed electron densities, averaged over these groups of three bands and over all *k*-points, further illustrate that the three highest-energy valence bands (top) are nonbonding and have almost entirely S character, while the three bands below those (bottom) are bonding and have both Bi *p* and S character.

Looking closely at these same bands, Figure 5 also supports the covalent view of this structure described in the previous subsection (i.e., 3-center 4-electron bonding). The three lowest-energy conduction bands have significant Bi *p* and S *p* character, the three highest-energy valence bands have primarily S *p* character, and the three valence bands below those have significant Bi *p* and S *p* character. As shown in Figure 5c, the compositions of these three groups of bands therefore carry the hallmarks of the three molecular orbitals in a 3-center 4-electron bond. Each group has three bands because each of the three X–B–X bond axes in RbBiS_2_ has this type of 3-center 4-electron bonding. To further make this point, images of the computed electron densities—averaged over these groups of three bands and over all *k*-points—are shown in Figure 5d. While these computed electron densities are understandably more complex than the schematic orbitals shown in Figure 5c, they support the presence of 3-center 4-electron bonding in RbBiS_2_. Consistent with the band structure and projected densities of states, the three highest-energy valence bands (Figure 5d, top) are nonbonding and have almost entirely sulfur character, while the three bands below those (Figure 5d, bottom) are bonding and have both bismuth *p* and sulfur character.

Yet more quantitative evidence for the differing covalent bonding in these three crystal structures is illustrated in Figure 6. This figure shows a region of the electron localization functions (with an isosurface value of 0.82) surrounding the B site of NaAsS_2_ in the three competing phases discussed throughout the paper. Essentially, the images show the B-site lone pair that the octet rule leads us to expect on an atom with three covalent bonds. When in trigonal pyramidal (left) or seesaw (center) coordination, the lone pair points away from its nearest neighbors. When in octahedral (right) coordination, the lone pair is centered at the B site. As lone pairs tend to point away from 2-center 2-electron covalent bonds, the placements of the lone pairs in the three distinct structures of NaAsS_2_ support our description of the bonding in each structure: 2-center 2-electron bonds in trigonal pyramidal coordination, 3-center 4-electron bonds in octahedral coordination, and both types of bonds in seesaw coordination.

**Figure 6:**
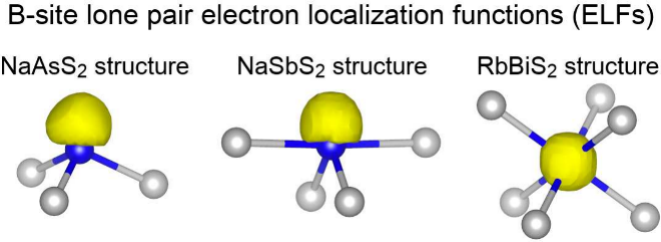
Electron localization functions (isosurface value = 0.82) surrounding the B site of NaAsS_2_ in the competing NaAsS_2_ (left)-, NaSbS_2_ (center)-, and RbBiS_2_-type (right) crystal phases. As lone pairs tend to point away from 2-center 2-electron covalent bonds, the placements of lone pairs imply the characterizations of B–X bonds described in the text.

### Relationships among Composition, Structure, and Band Gap

Figure 7 shows the band gaps of all combinations of elements in these three phases, computed using the hybrid HSE06 functional. For NaAsS_2_-type (Figure 7a), NaSbS_2_-type (Figure 7b), and RbBiS_2_-type (Figure 7c) phases, the taller dark bars show the computed gaps of sulfides and the shorter light bars show the computed gaps of selenides. Some trends are worth noting. As one would expect, the sulfide compounds generally have larger band gaps than the respective selenides, by an average of 0.37 ± 0.10 eV. Because sulfur is more electronegative than selenium and those elements contribute mainly to the valence bands, the valence bands of sulfides are lower in energy (and therefore the band gaps larger) than those of selenides. The trends in band gap with changing A-site composition are phase-dependent and interesting. One might expect that, because A-site orbitals do not contribute significantly to the band edges, A-site identity would not have much impact on the band gap. In RbBiS_2_-type compounds (Figure 7c), this is generally true, and band gaps are not significantly altered by changing the A-site element. In NaAsS_2_- (Figure 7a) and NaSbS_2_-type (Figure 7b) phases, however, the effect of A-site identity is dramatic, with larger A-site elements increasing the band gap by 1 or even 2 eV. This is because a large A-site element effectively separates the covalent B–X chains from each other, isolating them electronically by lengthening the more distant noncovalent interactions between B and X. Examples of this type of increase in band gap with larger A-site cations were pointed out in experimental work by Bera and co-workers on LiAsS_2_ and NaAsS_2_,^26^ and in computational work by Liu and co-workers on (Li,Na)­SbS_2_.^44^

**Figure 7:**
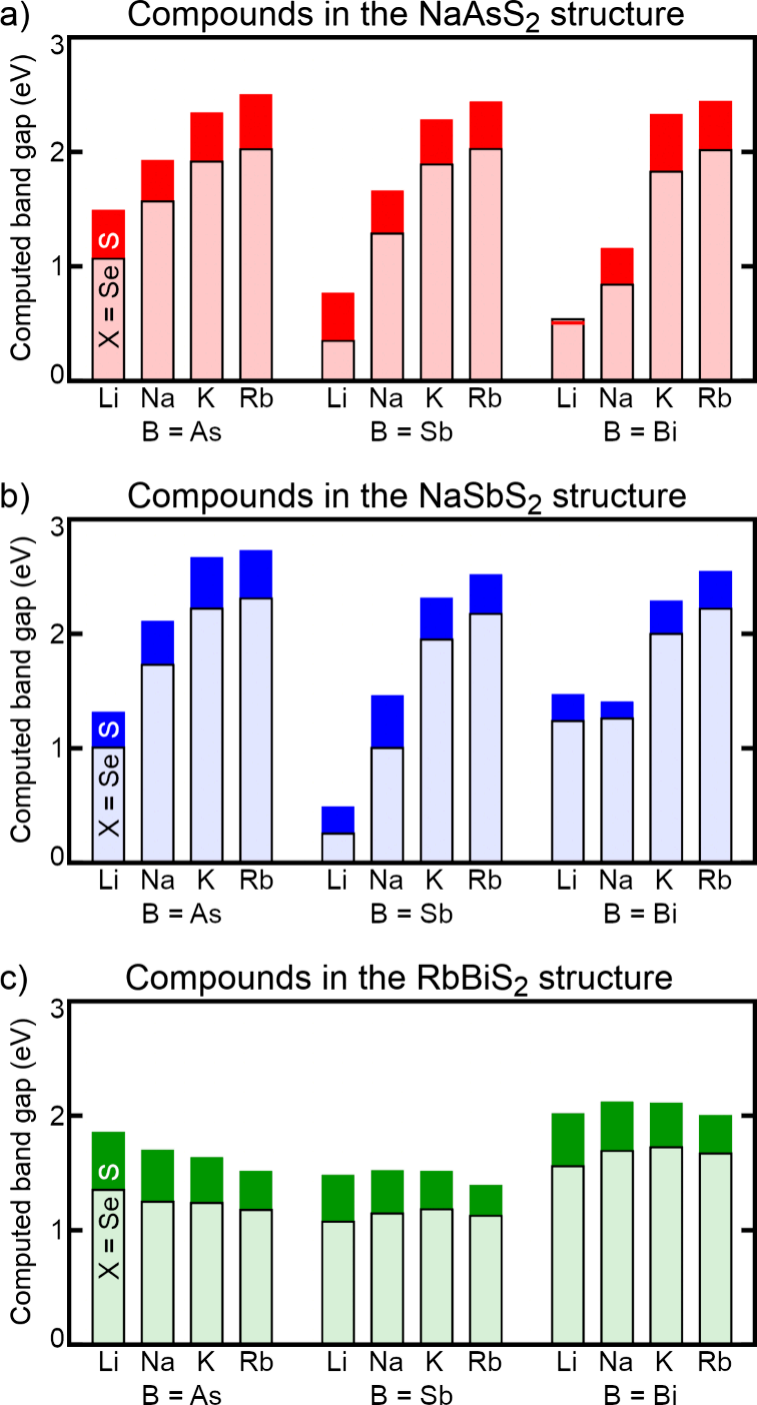
HSE06-computed band gaps of compounds in their geometry-optimized a) NaAsS_2_, b) NaSbS_2_, and c) RbBiS_2_ crystal phases. The taller dark bars show the band gaps of sulfides and the shorter light bars show the band gaps of the respective selenides.

Putting all of these energetic and band gap trends together, Figure 8 shows the computed band gaps of the lowest-energy structure of each combination of elements. Within this class of compounds, many are predicted to have band gaps in the most intense regions of the solar spectrum. Because Bi-containing compounds generally prefer the RbBiS_2_ structure, their band gaps are not significantly affected by the identity of A. In contrast, the band gaps of As- and Sb-containing compounds are highly tunable with changing A.

**Figure 8:**
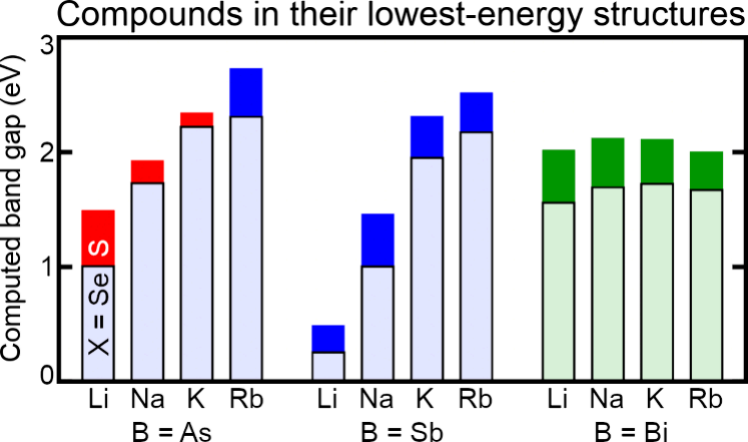
HSE06-computed band gaps of compounds in their geometry-optimized lowest-energy crystal phases. The taller dark bars show the band gaps of sulfides and the shorter light bars show the band gaps of the respective selenides. Bar colors are consistent with Figure 7.

## Conclusion

The atomic structure, chemical bonding, and electronic properties of the class of NaCl-type I–V–VI_2_ chalcogenide semiconductors described in this paper are notably diverse. Fundamentally, this diversity can be viewed as the result of a delicate balance of ionic and covalent behavior. As we have shown, the preferences for trigonal pyramidal, seesaw, or octahedral B-site coordination; 2-center 2-electron or 3-center 4-electron bonding; and degree of band gap tunability with A-site identity are all highly influenced by the degree of ionicity or covelence. Through the confluence of these factors, the band gaps among this class of compounds span the most intense regions of the solar spectrum (Figure 8). From a practical standpoint, this work enables the prediction and rational tuning of optoelectronic properties for solar energy conversion. For example, it suggests ways in which mixing elements at the A, B, and X sites or applying strains in particular directions could further access a range of band gaps.

## Supplementary Material



## Data Availability

The VASP input files (POSCAR, INCAR, and KPOINTS) are available on the authors’ research group GitHub page (https://github.com/bergerlab-wwu).

## Notes

Information about DFT pseudopotentials, tabulated numerical results corresponding to figures in the body of the paper, and further testing of the impact of number of *k*-points, basis set cutoff, and spin–orbit coupling on results (PDF)
