# Supplementary material for: Rationalizing Patterns in the Cation Ordering, Geometric Distortion, and Electronic Properties of a Class of I–V–VI2 Chalcogenide Semiconductors
Source: J Phys Chem C Nanomater Interfaces. 2026 Jan 12;130(3):1125–31. doi: 10.1021/acs.jpcc.5c05853 (PMC12833983; doi:10.1021/acs.jpcc.5c05853)
Supplement: Supplementary file 1 [file jp5c05853_si_001.pdf]

Supporting Information for "Rationalizing Patterns in the Cation Ordering, Geometric Distortion, and Electronic Properties of a Class of I–V–VI_2 Chalcogenide Semiconductors"

Gabe Flanagin and Robert F. Berger*

Department of Chemistry, Western Washington University, Bellingham WA 98225, USA

E-mail: bergerr@wwu.edu

VASP input files. For all calculations discussed in this paper, the VASP input files (POSCAR, INCAR, and KPOINTS) are available on the authors' research group GitHub page (https://github.com/bergerlab-wwu).

Pseudopotentials. For the PBE and HSE06 calculations performed using the VASP code throughout this paper, Table S1 shows which electrons are treated as valence in the PBE pseudopotentials.

Table S1: Electrons treated as valence in the VASP PBE pseudopotentials of each of the elements computed in this paper.

Site	Element	Valence electrons
A	Li	$2s^1$
	Na	$2p^6 3s^1$
	K	$3s^2 3p^6 4s^1$
	Rb	$4s^2 4p^6 5s^1$
B	As	$4s^2 4p^3$
	Sb	$5s^2 5p^3$
	Bi	$5d^{10} 6s^2 6p^3$
X	S	$3s^2 3p^4$
	Se	$4s^2 4p^4$

Tabulated results from body of paper. Tables S2 and S3 show the DFT-PBE-computed energies of optimized structures and DFT-HSE06-computed band gaps reported in the body of the paper.

Table S2: Comparisons of the DFT-PBE-computed structural energy per atom of compounds in their geometry-optimized $NaAsS_2$, $NaSbS_2$, and $RbBiS_2$ crystal phases, as in Figure 2 in the body of the paper. For each combination of elements, the structural energy per atom of the $RbBiS_2$ structure is defined to be the zero-energy baseline.

A	B	X	$NaAsS_2$ phase energy/atom (eV)	$NaSbS_2$ phase energy/atom (eV)	$RbBiS_2$ phase energy/atom (eV)
Li	As	S	−0.0483	−0.0469	≡ 0
Na			−0.0779	−0.0707	
K			−0.1244	−0.1236	
Rb			−0.1545	−0.1588	
Li	Sb	S	+0.0016	−0.0197	≡ 0
Na			−0.0167	−0.0349	
K			−0.0356	−0.0581	
Rb			−0.0515	−0.0791	
Li	Bi	S	+0.0636	+0.0158	≡ 0
Na			+0.0680	+0.0236	
K			+0.0842	+0.0375	
Rb			+0.0797	+0.0291	
Li	As	Se	−0.0204	−0.0244	≡ 0
Na			−0.0333	−0.0367	
K			−0.0640	−0.0745	
Rb			−0.0865	−0.1028	
Li	Sb	Se	+0.0190	−0.0018	≡ 0
Na			+0.0118	−0.0102	
K			+0.0043	−0.0224	
Rb			−0.0066	−0.0384	
Li	Bi	Se	+0.0746	+0.0297	≡ 0
Na			+0.0771	+0.0332	
K			+0.0993	+0.0485	
Rb			+0.0979	+0.0425	

Table S3: HSE06-computed band gaps of compounds in their DFT-PBE-optimized $NaAsS_2$, $NaSbS_2$, and $RbBiS_2$ crystal phases, as in Figure 7 in the body of the paper. These band gaps are computed without spin-orbit coupling. See Tables S4 and S5 for comparisons to other methods.

A	B	X	$NaAsS_2$ phase band gap (eV)	$NaSbS_2$ phase band gap (eV)	$RbBiS_2$ phase band gap (eV)
Li	As	S	1.4944	1.3199	1.8599
Na			1.9307	2.1132	1.7026
K			2.3447	2.6694	1.6388
Rb			2.5035	2.7301	1.5159
Li	Sb	S	0.7682	0.4909	1.4840
Na			1.6623	1.4641	1.5252
K			2.2835	2.3158	1.5182
Rb			2.4388	2.5212	1.3947
Li	Bi	S	0.5207	1.4729	2.0217
Na			1.1594	1.4093	2.1253
K			2.3309	2.2917	2.1136
Rb			2.4447	2.5478	2.0049
Li	As	Se	1.0664	1.0047	1.3511
Na			1.5676	1.7280	1.2476
K			1.9152	2.2204	1.2376
Rb			2.0214	2.3079	1.1748
Li	Sb	Se	0.3481	0.2546	1.0740
Na			1.2861	1.0035	1.1452
K			1.8896	1.9492	1.1821
Rb			2.0240	2.1726	1.1257
Li	Bi	Se	0.5391	1.2382	1.5595
Na			0.8396	1.2584	1.6926
K			1.8256	1.9983	1.7242
Rb			2.0141	2.2188	1.6702

Additional tabulated results. For comparison to the results reported in the body of the paper, Tables S4 and S5 show band gaps computed using different methods (which lead to qualitatively similar trends), and Table S6 shows that structural energies and band gaps do not change significantly when the number of k-points and plane-wave basis set cutoff increase, or when van der Waals corrections are included.

Table S4: DFT-PBE-computed band gaps of compounds in their DFT-PBE-optimized $NaAsS_2$, $NaSbS_2$, and $RbBiS_2$ crystal phases. These DFT-PBE-computed band gaps are (as expected) smaller than the corresponding values computed with hybrid functionals, but show qualitatively similar trends.

A	B	X	$NaAsS_2$ phase band gap (eV)	$NaSbS_2$ phase band gap (eV)	$RbBiS_2$ phase band gap (eV)
Li	As	S	0.9790	0.7794	1.2584
Na			1.2966	1.4110	1.1082
K			1.5995	1.9019	1.0562
Rb			1.7312	1.9761	0.9461
Li	Sb	S	0.4987	0.1172	0.9781
Na			1.1187	0.8707	0.9630
K			1.6014	1.6072	0.9591
Rb			1.7236	1.7965	0.8453
Li	Bi	S	0.2003	1.0932	1.4128
Na			0.7356	0.9844	1.4561
K			1.5717	1.6388	1.4474
Rb			1.7342	1.8558	1.3510
Li	As	Se	0.6081	0.6693	0.8220
Na			1.0266	1.1140	0.7252
K			1.2742	1.4744	0.7267
Rb			1.3554	1.5678	0.6699
Li	Sb	Se	0.2585	0.1795	0.6182
Na			0.8514	0.5433	0.6393
K			1.3104	1.3307	0.6849
Rb			1.4026	1.5314	0.6311
Li	Bi	Se	0.2377	0.8352	1.0138
Na			0.4891	0.8895	1.0877
K			1.1935	1.3500	1.1249
Rb			1.3504	1.5040	1.0753

Table S5: HSE06-computed band gaps of compounds in their DFT-PBE-optimized $NaAsS_2$, $NaSbS_2$, and $RbBiS_2$ crystal phases, including spin-orbit coupling. In addition to the inclusion of spin-orbit coupling, these calculations also differ from those presented in the body of the paper (and Table S3) in that they use half the number of k-points in each dimension for reasons of computational feasibility. Once again, band gap trends are qualitatively similar to those computed using other methods.

A	B	X	$NaAsS_2$ phase band gap (eV)	$NaSbS_2$ phase band gap (eV)	$RbBiS_2$ phase band gap (eV)
Li	As	S	1.6030	1.3185	1.8871
Na			2.0625	2.1086	1.7589
K			2.4274	2.6640	1.6906
Rb			2.5735	2.7384	1.5919
Li	Sb	S	1.3795	0.4908	1.6259
Na			1.7963	1.4298	1.5439
K			2.3704	2.2805	1.5272
Rb			2.5040	2.4814	1.4373
Li	Bi	S	1.6163	0.7076	1.7136
Na			1.6275	0.7459	1.7112
K			2.2757	1.9634	1.7440
Rb			2.3744	2.2305	1.6685
Li	As	Se	1.1579	0.9936	1.4331
Na			1.6023	1.7074	1.3295
K			1.9392	2.2490	1.2960
Rb			2.0377	2.3301	1.2248
Li	Sb	Se	0.9705	0.3069	1.2765
Na			1.3904	0.9594	1.2001
K			1.9166	1.9012	1.2216
Rb			2.0307	2.1255	1.1514
Li	Bi	Se	1.2519	0.5528	1.3015
Na			1.2183	0.6385	1.3207
K			1.6860	1.6965	1.3579
Rb			1.8511	1.9349	1.3280

Table S6: Comparisons of the DFT-PBE-computed structural energy per atom and band gap of $NaAsS_2$, $NaSbS_2$, and $RbBiS_2$ in all crystal phases, with various input parameters and methods to test consistency. "Baseline" calculations refer to those described in the Methods section of the paper (k-points matching a cubic NaCl-type unit cell with a $6 \times 6 \times 6$ mesh, and a basis set cutoff of 400 eV). Other columns have approximately 50% more k-points in each dimension, a basis set cutoff of 450 eV, or van der Waals corrections using the D3 method of Grimme.

Stoichiometry = $NaAsS_2$	Baseline	More k-points	Higher cutoff	vdW
$NaAsS_2$ phase energy/atom (eV)	−0.0779	−0.0779	−0.0781	−0.0519
$NaSbS_2$ phase energy/atom (eV)	−0.0707	−0.0707	−0.0708	−0.0524
$RbBiS_2$ phase energy/atom (eV)	$\equiv 0$	$\equiv 0$	$\equiv 0$	$\equiv 0$
$NaAsS_2$ band gap (eV)	1.2966	1.2953	1.2939	1.2563
$NaSbS_2$ band gap (eV)	1.4110	1.4187	1.4138	1.2392
$RbBiS_2$ band gap (eV)	1.1082	1.1187	1.1083	1.1383
Stoichiometry = $NaSbS_2$	Baseline	More k-points	Higher cutoff	vdW
$NaAsS_2$ phase energy/atom (eV)	−0.0167	−0.0166	−0.0168	−0.0062
$NaSbS_2$ phase energy/atom (eV)	−0.0349	−0.0349	−0.0349	−0.0281
$RbBiS_2$ phase energy/atom (eV)	$\equiv 0$	$\equiv 0$	$\equiv 0$	$\equiv 0$
$NaAsS_2$ band gap (eV)	1.1187	1.0870	1.1222	0.9917
$NaSbS_2$ band gap (eV)	0.8707	0.8703	0.8757	0.6835
$RbBiS_2$ band gap (eV)	0.9630	0.9764	0.9620	0.9804
Stoichiometry = $RbBiS_2$	Baseline	More k-points	Higher cutoff	vdW
$NaAsS_2$ phase energy/atom (eV)	+0.0797	+0.0798	+0.0797	+0.1025
$NaSbS_2$ phase energy/atom (eV)	+0.0291	+0.0291	+0.0291	+0.0492
$RbBiS_2$ phase energy/atom (eV)	$\equiv 0$	$\equiv 0$	$\equiv 0$	$\equiv 0$
$NaAsS_2$ band gap (eV)	1.7342	1.7331	1.7343	1.6210
$NaSbS_2$ band gap (eV)	1.8558	1.8541	1.8546	1.7669
$RbBiS_2$ band gap (eV)	1.3510	1.3594	1.3494	1.3889
